# Evidence of HIV exposure and transient seroreactivity in archived HIV-negative severe hemophiliac sera

**DOI:** 10.1186/1743-422X-2-65

**Published:** 2005-08-17

**Authors:** Scott A Tenenbaum, Cindy A Morris, Steve S Alexander, Harris E McFerrin, Robert F Garry, Cindy A Leissinger

**Affiliations:** 1 Department of Biomedical Sciences, Ge*NY*Sis Center for Excellence in Cancer Genomics, University at Albany-SUNY, Albany, NY, USA; 2 Microbiology and Immunology, Tulane University School of Medicine, Tulane University School of Medicine, New Orleans, LA, USA; 3 Ortho Diagnostic Systems, HlV and Hepatitis Research and Development, Raritan, NJ, USA; 4 Department of Medicine, Section of Hematology and Medical Oncology, Tulane University School of Medicine, New Orleans, LA, USA

## Abstract

**Background:**

Approximately 25% of hemophiliacs that were frequently exposed to blood clotting factor concentrates (CFCs) contaminated with human immunodeficiency virus (HIV) are presently HIV seronegative. In this study, we sought to determine if some of these individuals were at any time transiently HIV seropositive. In the early to mid-1980s the majority of severe hemophilia patients were exposed to CFCs contaminated with HIV. Although many of these hemophiliacs became HIV-positive, a small percentage did not become infected. To determine if some of these individuals successfully resisted viral infection, we attempted to document the presence of transient HIV reactive antibodies in archived plasma samples (1980–1992) from currently HIV-negative severe hemophiliacs who had a high probability of repeated exposure to HIV contaminated CFC. Archived plasma samples were retrospectively tested using an FDA approved HIV-1Ab HIV-1/HIV-2 (rDNA) enzyme immunoassay (EIA) and a HIV-1 Western blot assay (Wb), neither of which were commercially available until the late 1980s, which was after many of these samples had been drawn.

**Results:**

We found that during the high risk years of exposure to HIV contaminated CFC (1980–1987), low levels of plasma antibodies reactive with HIV proteins were detectable in 87% (13/15) of the haemophiliacs tested. None of these individuals are presently positive for HIV proviral DNA as assessed by polymerase chain reaction (PCR).

**Conclusion:**

Our data suggest that some severe hemophiliacs with heavy exposure to infectious HIV contaminated CFC had only transient low-level humoral immune responses reactive with HIV antigens yet remained HIV-negative and apparently uninfected. Our data supports the possibility of HIV exposure without sustained infection and the existence of HIV-natural resistance in some individuals.

## Background

In the 1980's an estimated 17,000 people in the United States were affected the congenital blood clotting factor deficiencies, Hemophilia A and B (Factor VIII and Factor IX deficiency, respectively). Since the early 1970's, the mainstay of treatment for bleeding in hemophilia patients has been the use of clotting factor concentrates (CFCs) commercially prepared from large plasma pools comprised of thousands of individual donors. Prior to 1985 CFCs were prepared from donors with unknown HIV infection status and were not routinely subjected to viral inactivation procedures. With each infusion from a new lot of clotting factor concentrate, hemophilia patients were exposed to plasma from approximately 2,000 to 25,000 donors [1]. As a result, roughly 50% of the total hemophilia population in the United States became infected with HIV prior to the institution of donor screening and the use of viral inactivation procedures of factor concentrates in 1985 [2-4]. Since 1987 there has been a virtual elimination of HIV-1 infection in the hemophilia population [3-6].

Largely due to the extensive network of comprehensive hemophilia treatment centers, the hemophilia population has been actively studied for possible variables that may influence HIV infection and progression. Retrospective analysis of hemophiliac plasma samples stored as part of routine clinical visits has shown that HIV infection, as documented by permanent HIV-seroconversions began in 1978, peaked in 1982, and ended by 1987. In general, those patients who received the greatest exposure to CFCs were at the highest risk for HIV infection [7]. Hemophiliacs exposed to factor-VIII concentrates, in general, were more likely to become infected than those exposed to factor-IX concentrates (prothrombin complex concentrates or PCCs). Patients who received an average of over 20,000 units of factor-VIII concentrate annually during the early 1980's had a cumulative incidence of HIV-infection exceeding 90% and those receiving comparable doses of PCCs had a cumulative incidence exceeding 50% [3,4]. This clearly demonstrates the prevalence of infectious HIV in the United States CFC supply.

Not all hemophiliacs exposed to CFCs contaminated with infectious HIV were ultimately infected with the HIV virus. Although inoculum size may account for the lack of infection in some hemophiliacs, factors such as age, race, sex or pre-existing medical condition has not been found to be related to risk of HIV infection. However, several studies have shown that certain HLA types were associated with either an increased or decreased risk of HIV infection in hemophilia patients [3,8-10].

In 1996, three independent groups identified the chemokine co-receptor 5 (CCR-5) as a secondary receptor for the HIV virus. The presence of two copies of a naturally occurring deletion mutation of the CCR-5 receptor (CCR-5Δ32) apparently conferred resistance to infection by the virus [11-14]. Heterozygous expression of CCR-5Δ32 did not appear to prevent HIV-1 infection but may have resulted in slower decline in CD4+ cells, lower levels of plasma viremia and in slower progression to AIDS [15-18]. From 1979–1985 severe hemophiliacs, as defined as individuals producing less than 1% of the normal value of a clotting factor, were exposed to the largest volume of clotting factor concentrates. Accordingly, 90% of these individuals became infected with HIV [4]. However, some severe hemophiliacs have remained H [V-negative despite repeated exposure to CFCs. We hypothesized that information concerning natural HIV resistance might be obtained by the investigation of such high-risk individuals. Although HIV enzyme linked immunoassays (EIA) and Western blot assays (Wb) were available for detecting antibodies reactive with HIV antigens as early as 1984, the specificities and sensitivities of these immunoassays have increased dramatically in more contemporary versions of these diagnostic tests [19-23]. We speculated that some High-risk hemophiliacs exposed to contaminated CFCs, although not permanently infected, may have mounted transient humoral and/or cellular immune responses reactive with HIV proteins but were below the threshold of delectability of the earlier first generation HIV diagnostic tests. Using more sensitive HIV immunologic diagnostic tests, we reassessed anti-HIV reactivity in archived plasma samples (1980–1992) from presently HIV-seronegative severe hemophiliacs that had exposure to large quantities of contaminated CFCs (HIV exposed/-HIV negative hemophiliacs).

## Results

### Detection of transient HIV reactive plasma antibodies

**Figure 1:**
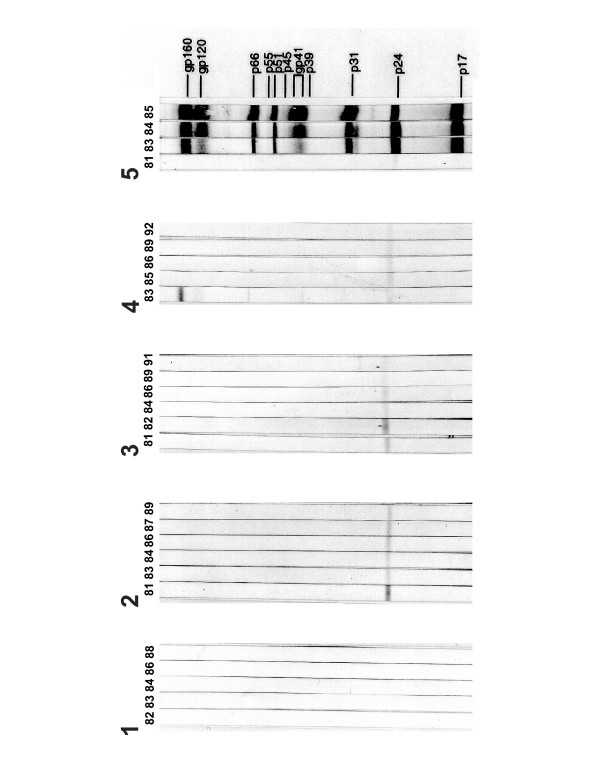
**HIV-1 Western blot reactivity of HIV-exposed/HIV seronegative hemophiliacs. **Panels 1–4: HIV-1 western blot analysis of chronologically archived plasma samples from HIV-exposed/HIV-seronegative hemophiliacs displaying transient partial reactivity against HIV-1 proteins. Panel 5: Chronological serum samples from an HIV-1 seropositive hemophiliac. Panel numbers (1–4) correspond to 1–4 respectively in tables 1 and 2.

We had adequate yearly representation and sample quantity in our archived collection to assemble plasma sets from 15 severe hemophiliacs with extremely likely exposure to HIV contaminated clotting concentrates (Table 1). All of the archived plasma sets tested contained samples that were collected prior to 1986. Using improved HIV-EIA or HIV-Wb immunoassay analysis we detected antibodies reactive with HIV antigens in one or more samples from 13/15 (87%) of the archived plasma sets tested (Figure 1, panels 2-4 and Table 2, patients 1–13). Of these, two plasma sets had samples reactive to both the HIV-EIA and the HIV-WB (Figure 1, panel 2 and Table 2, patient 2 and 5). An additional three plasma sets contained samples displaying reactivity only on the HIV-EIA (Table 2, patients 1, 6 and 7) which occurred in samples collected in 1987 or earlier. Eight of the hemophilia patient archived plasma sets contained samples with antibodies (IgG and/or IgM) only reactive with one or more HIV antigen as determined by HIV-1 Wb analysis (representative examples panels 2–4, Figure 1 and Table 2, patients 3, 4, 8–13). HIV proteins were recognized by antibodies in HIV-1 Wb reactive plasma samples in the following frequencies; group antigen (Gag) p17 (3/10, 30%), group antigen (Gag) p24 (8/10, 80%), reverse transcriptase (RT) p66 (3/10, 30%), envelope glycoproteins (Env) gp120 and 160 (2/10, 20%), One archived plasma set contained a sample that displayed reactivity to multiple HIV antigens on HIV-1 Wb analysis that approached meeting the criteria for "HIV-positive" reactivity (Figure 1, panel 4 and table 2, patient 4). The observed multiple HIV protein reactivity was only present in the 1983 sample from this archived plasma set. As a whole, indeterminate banding activity on the HIV-1 Wb appeared to fluctuate in intensity within many of the archived plasma sets that displayed reactivity (Figure 1 panels 1–3 and Table 2 asterisks). The greatest intensity of banding activity in these archived plasma sets coincided with years of highest-risk of exposure to HIV contaminated clotting factor concentrate (1980–1985). None of the HIV-1 Wb indeterminate hemophiliacs showed reactivity to HIV-2 proteins when analyzed by HIV-2 Wb analysis (data not shown).

**Table 1:** Clinical profile and clotting factor concentrate exposure of HIV-Exposed/HIVseronegative hemophiliacs.

		Hemophilia			Factor_concentrate exposure (1980–1985)	
Patient #	Year of birth	Type	Severity*	Presence of inhibitor**	Type	Average Units/year
1	1972	B	<1%	No	PCC	55,000
2	1956	B	4%	No	PCC	<10,000
3	1966	A	<1%	Yes	PCC	27,000
4	1953	A	<1%	No	VIII	15,000
5	1977	B	<1%	No	PCC	29,000
6	1971	A	<1%	Yes	PCC	30,000
7	1931	A	<1%	No	VIII	11,000
8	1976	B	<1%	No	PCC	18,000
9	1964	B	<1%	No	PCC	24,000
10	1976	A	<1%	Yes	PCC	12,000
11	1957	A	<1%	No	VIII	29,000
12	1950	A	<1%	Yes	PCC	80,000
13	1969	B	2%	No	PCC	22,000
14	1959	A	<1%	No	VIII	10,000
15	1946	B	<1%	No	PCC	19,000

* Normal factor VIII and Ix levels are 50–150%

** Inhibitor indicates the presence of a circulating antibody against the deficient factor; for patients with hemophilia A, an inhibitor often necessitates the use of PCC instead of factor VIII concentrates to control bleeding.

**Table 2:** Transient HIV-1 Seroactivity in HIV-Exposed Hemophiliacs

Plasma Draw		HIV-EIA	HIV-Western blot IgG					HIV-Western blot IgM					**CCR5
Patient#	Date	^1^S:C	p17	p24	p66	gpl20	gpl60	p17	p24	p66	gpl20	gpl60	Deletion
1	82	0	-	-	-	-	-	-	-	-	-	-	+/-
	83	1.065	-	-	-	-	-	-	-	-	-	-	
	84	0	-	-	-	-	-	-	-	-	-	-	
	86	0	-	-	-	-	-	-	-	-	-	-	
	88	0	-	-	-	-	-	-	-	-	-	-	
													
2	81	0	-	+*	-	-	-	-	-	+	-	-	ND
	83	0	-	+	-	-	-	-	-	+	-	-	
	84	0	-	+	-	-	-	-	-	+	-	-	
	86	1.143	-	+	-	-	-	-	-	+	-	-	
	87	0	-	+	-	-	-	-	-	+	-	-	
	89	0	-	+	-	-	-	-	-	+	-	-	
													
3	81	0	-	+	-	-	-	-	+	-	-	-	+/+
	82	0	-	+*	-	-	-	-	+	-	-	-	
	84	0	-	+	-	-	-	-	+	-	-	-	
	86	0	-	+	-	-	-	-	+	-	-	-	
	89	0	-	±	-	-	-	-	+	-	-	-	
	91	0	-	±	-	-	-	-	+	-	-	-	
													
4	83	0	-	+	+	+	+*	-	+	+	+	+	+/+
	85	0	-	+	-	-	-	-	-	-	-	-	
	86	0	-	+	-	-	-	-	-	-	-	-	
	89	0	-	+	-	-	-	-	-	-	-	-	
	92	0	-	+	-	-	-	-	-	-	-	-	
													
5	80	0	-	-	-	-	-	-	-	-	-	-	+/+
	81	2.437	-	+	+	-	-	-	-	-	-	-	
	85	0	-	+	+	-	-	-	-	-	-	-	
	86	0	-	+	+	-	-	-	-	-	-	-	
	87	0		+	+	-	+	-	-	-	-	-	
	88	0	-	-	-	-	-	-	-	-	-	-	
	90	0	-	-	-	-	-	-	-	-	-	-	
	92	0	-	-	-	-	-	-	-	-	-	-	
													
6	81	0	-	-	-	-	-	-	-	-	-	-	+/+
	82	0	-	-	-	-	-	-	-	-	-	-	
	85	1.519	-	-	-	-	-	-	-	-	-	-	
	86	1.104	-	-	-	-	-	-	-	-	-	-	
	87	1.117	-	-	-	-	-	-	-	-	-	-	
	88	0	-	-	-	-	-	-	-	-	-	-	
													
7	82	1.143	-	-	-	-	-	-	-	-	-	-	+/+
	83	0	-	-	-	-	-	-	-	-	-	-	
	86	0	-	-	-	-	-	-	-	-	-	-	
													
8	82	0	-	+	-	-	-	+*	+	-	-	-	+/+
	84	0	-	+	-	-	-	+	+	-	-	-	
	85	0	-	+	-	-	-	+	+	-	-	-	
	86	0	-	+	-	-	-	±	±	-	-	-	
	92	0	-	+	-	-	-	±	±	-	-	-	
													
9	80	0	-	+	-	-	-	±	+	-	-	-	+/+
	81	0	-	+	-	-	-	+	+	-	-	-	
	82	0	-	+*	-	-	-	+*	+	-	-	-	
	85	0	-	+	-	-	-	+	+	-	-	-	
	87	0	-	+	-	-	-	+	+	-	-	-	
	92	0	-	+	-	-	-	+	+	-	-	-	
													
10	81	0	-	+	-	-	-	+	+	-	-	-	+/+
	84	0	-	+	-	-	-	+	+	-	-	-	
	85	0	-	+	-	-	-	+	+	-	-	-	
	86	0	-	+	-	-	-	+	+	-	-	-	
	89	0	-	+	-	-	-	+	+	-	-	-	
	90	0	-	+	-	-	-	+	+	-	-	-	
	92	0	-	+	-	-	-	+	+	-	-	-	
													
11	85	0	-	-	-	-	-	-	+	-	-	-	-/-
	86	0	-	-	-	-	-	-	+	-	-	-	
	87	0	-	-	-	-	-	-	+	-	-	-	
	89	0	-	-	-	-	-	-	+	-	-	-	
													
12	83	0	-	-	±	-	-	-	-	-	-	-	+/+
	84	0	-	-	±	-	-	-	-	-	-	-	
	85	0	-	-	±	-	-	-	-	-	-	-	
	87	0	-	-	±	-	-	-		-	-	-	
	91	0	-	-	±	-	-	-	-	-	-	-	
													
13	81	0	-	+	-	-	-	-	±	-	-	-	+/+
	82	0	-	+	-	-	-	-	±	-	-	-	
	83	0	-	+	-	-	-	-	±	-	-	-	
	84	0	-	+	-	-	-	-	±	-	-	-	
	85	0	-	+	-	-	-	-	±	-	-	-	
	87	0	-	+	-	-	-	-	±	-	-	-	
	91	0	-	+	-	-	-	-	±	-	-	-	
													
14	80	0	-	-	-	-	-	-	-	-	-	-	+/-
	82	0	-	-	-	-	-	-	-	-	-	-	
	84	0	-	-	-	-	-	-	-	-	-	-	
	85	0	-	-	-	-	-	-	-	-	-	-	
	86	0	-	-	-	-	-	-	-	-	-	-	
	92	0	-	-	-	-	-	-	-	-	-	-	
													
15	83	0	-	-	-	-	-	-	-	-	-	-	+/+
	84	0	-	-	-	-	-	-	-	-	-	-	
	85	0	-	-	-	-	-	-	-	-	-	-	
	86	0	-	-	-	-	-	-	-	-	-	-	
	91	0	-	-	-	-	-	-	-	-	-	-	

^1^S:C Denotes the ratio of sample absorbance value to cut-off value.

*Sample displaying the most reactivity in archived plasma set with fluctuating anti-HIV-l antibody reactivity on WB analysis.

**CCR5 Deletion Analysis

ND = not done

+/+ = wildtype,

+/- = mutant heterozygous

-/- = mutant homozygous

### Assessment of current HIV-1-proviral DNA status

Post-1990 peripheral blood mononuclear cells were assessed for the presence of HIV-1 proviral DNA in all patients whose samples showed anti-HIV-1 reactivity using HIV-1-PCR analysis (kindly performed in a blinded manner using appropriate positive and negative controls by Charles Schable at the Centers for Disease Control and Prevention, Atlanta, GA.) All currently seronegative patients were found to be negative for HIV-1 proviral DNA by PCR analysis (data not shown).

### Passive HIV-1 reactive antibodies in clotting Factor concentrates

It was possible that the HIV-1 activity that we detected results from the presence of passive anti-HIV antibodies that were present in a recently transfused CFC just prior to the drawing of the plasma sample we tested and therefore could be detectable in the plasma sample we tested. To assess the feasibility that the HIV-1 reactive antibodies present in contaminated CFCs could passively give rise to false-positive results in our testing, we reconstituted four factor-VIII concentrates and one PCC that were manufactured between 1981 and 1984 and tested them for the presence of anti-HIV-1 reactive antibodies. None of the reconstituted concentrates displayed reactivity on HIV-EIA, although a control aliquot of reconstituted factor spiked with HIV positive serum was reactive (data not shown) suggesting that the observed HIV-1 reactivity on our assays was not the result of the passive presence of HIV-1 reactive antibodies in CFCs. The reconstituted PCC was negative by HIV-1 WB analysis, however, the reconstituted factor VIII lots were found to have HIV-1 Wb reactivity at a dilution of 1:50 for the viral antigens p17, p24 and gpl60. One reconstituted factor HIV-1 sample had detectable anti-HIV-1 reactivity at a dilution of 1:100 (which was the dilution used for the hemophiliac plasma samples) but not beyond. No HIV-1 EIA or Wb reactivity was detected in clotting factor concentrates manufactured after 1985 (data not shown).

### Presence of CCR-5 deletion mutations

To determine whether hetero- or homozygous CCR-5 deletion mutations were present in these patients, polymerase chain reaction-restriction fragment length polymorphism (PCR-RFLP) analyses were performed on samples obtained from all patients except patient 2 (Table 2). As expected most patients (11/14) were homozygous for wild type CCR -5. 2 of 14 patients were heterozygous for CCR-5Δ32. Interestingly, patient 11, who was homozygous positive for the mutation, was never infected; however, he was the most heavily exposed to the most infectious concentrate (factor VIII) and was an IV drug user. Because our laboratory has been following longitudinally a number of hemophiliacs (113) who have been exposed to CFCs, including the 15 patients followed throughout this study, we determined the CCR5 genotypes of these individuals to establish the frequency of CCR5 mutations. Expectedly, the vast majority of the hemophiliacs tested (113) were wild-type for CCR5; however, 14/131 (10.7%) and 2/131 (1.5%) were heterozygous and homozygous mutants of CCR5, respectively. Of these patients, 47 were HIV-1-positive, where 41/47 (87.2%), 6/47 (12.8%) and 0/47 (0%) were homozygous wild-type, heterozygous and homozygous mutant for CCR5, in that order. Of the remaining patients that were HIV-1-negative genotyped (a total of 84), 74/84 (88.1%), 8/84 (9.5%) and 2/84 (2.4%) were homozygous wild-type, heterozygous and homozygous mutant for CCR5 respectively.

## Discussion

The presence of antibodies with specificity for multiple HIV-1 proteins is one of the diagnostic hallmarks of infection with the HIV-1 virus. In virtually all patients infected with HIV-1 permanent anti-viral antibodies are detectable typically within 3 to 12 weeks following exposure to the virus, and continue to increase in titer during the early phase of infection. Following this early phase, anti-HIV-1 antibody titer generally remains constant until the end stages of AIDS when fluctuations in total anti-HIV immunoglobulin may occur. Several studies have attempted to evaluate the existence of individuals who have been at high-risk of exposure to presumably infectious HIV, but who have resisted HIV-1 infection. This work has focused on several populations of high-risk HIV-1 seronegative individuals including intravenous drug users [24-26], HIV-1 exposed health care workers [27-29], sexual partners of HIV-1 infected individuals [27,30-40], female sex workers who have engaged in repeated unprotected sex with HIV-1 infected partners [30-32,38-44], HIV-1 uninfected children born from HIV-1 infected mothers [31,39,40,45,46] and hemophiliacs with a high probability of HIV-1 exposure from contaminated clotting factor concentrates [7,47-50].

In this study we retrospectively identified transient anti-HIV-1 antibody reactivity in archived plasma sets from currently HIV seronegative hemophiliacs who had a high probability of intravenous exposure to HIV contaminated CFCs. To accomplish this we used diagnostic methods for the detection of HIV reactive antibodies that are substantially more sensitive than earlier versions that were first introduction in the mid-80s when many of our plasma samples were originally tested and found to be negative. Confirmation of the current negative HIV status of the hemophiliacs whose archived samples were analyzed in this study was accomplished using HIV-l PCR analysis on recently obtained PBMC from these individuals.

Using HIV-EIA analysis we found that 5/15 (33%) archived plasma sets contained samples transiently reactive at one or more time point in 1987 or before. HIV-EIA reactivity above baseline is seen in less than 0.2% of healthy blood donors with no known exposure to HIV (HIV-1/HIV-2 EIA packet insert, Abbott Laboratories). One interpretation of these results is that there was a temporary appearance of low level antibodies reactive with HIV antigens in some hemophiliacs during, or shortly after, the most likely time of exposure to infectious or non-infectious HIV-1 in contaminated CFCs (1980–1985). The level of reactivity that we detected on the HIV-EIA was clearly above the baseline cut-off values determined by the respective controls for the assay, although lower than that typically seen in HIV-1 seropositive individuals.

None of the samples that were reactive on the HIV EIA showed reactivity against HIV-2 proteins by WB analysis suggesting that the HIV-2 antigens present on the EIA assay (Abbott Laboratories) were not likely responsible for the observed reactivity. The lack of anti-HIV-1 reactivity in samples older than those displaying reactivity suggested that these observations were not an artefact of the prolonged frozen storage of the plasma (Table 2, patient 1, 2 and 4–7).

Of the archived plasma sets that we tested, 10/15 (75%) had detectable IgG and/or IgM antibodies reactive with one or more HIV antigen on HIV-Wb analysis, which was most commonly directed against the p24 group antigen (figure 1 and table 2). Although most of the Wb reactivity that we observed would be classified as HIV-1 indeterminate, one archived plasma sample set had fluctuating weak IgG reactivity against multiple HIV proteins which included p24, p66 and gp160. This individual was also reactive by HIV-EIA analysis but only in one year corresponding to Wb activity (table 2, patient 5). A second hemophiliac, not reactive by HIV-EIA analysis, also had plasma antibodies reactive to HIV p24, p66, gp120 and gp160 antigens (Fig. 1, panel 4 and Table 2 patient 4). The p24 reactivity observed in this archived plasma set was consistently present in all of samples, however, the p66, gp120 and gp160 reactivity was observed only in the 1983 sample. Fluctuating anti-HIV reactive antibody titer was noted in samples from 5/10 (50%) of the archived plasma sets (Fig. 1 strip set 2–4 and Table 2, asterisks by patients 2–4, 8 and 9).

Indeterminate HIV-1 WB reactivity has been typically detected in only 5–7% of healthy EIA negative controls [51]. Although indeterminate results occurring in up to 32% of low-risk healthy populations have been reported with approximately half of these being attributable to p24 reactivity [52]. However, in our study, we observed that 10/15 (67%) had indeterminate reactivity in the archived plasma sets that we tested from hemophiliacs exposed to HIV-1 contaminated CFCs. Not surprisingly, reactivity to the p24 group antigen was the most frequent pattern that we observed. Most individuals when exposed to infectious HIV-1 will develop anti-gp120/160 envelope antibodies in addition to the p24 core antigen. In contrast, exposure to lysed HIV virus results almost exclusively in an anti-p24 response (Steve Alexander, personnel observations). Based on the pattern of HIV-Wb reactivity observed in the archived plasma sets tested in this study, our population of hemophiliacs were likely exposed predominantly to inactivated HIV-1. It is also possible that patterns of Wb reactivity more consistent with exposure to infectious HlV-1 may have existed in the hemophiliacs that we studied but was at a time not available in our archived collection of samples.

Among the five archived plasma sets that contained samples displaying HIV-EIA reactivity, only two (40%) also displayed HIV-Wb reactivity, which was not necessarily in corresponding years (Table 2., patients 2 and 4). Discrepancies between these two assays have previously been noted [53,54]. The HIV-Wb assay is an extremely sensitive method for the detection of antibodies which recognize predominantly, if not exclusively linearized epitopes. We have typically been able to detect anti-HIV serum antibodies from infected individuals when diluted more than a million fold. It is possible that the low level of reactivity noted in our archived plasma sets on HIV-Wb analysis may frequently have been beneath the limit of delectability by HIV-EIA. Discrepancies in reactivity between these two immunodiagnostic methods may also have resulted from the potential availability of conformational viral epitopes present on the HIV-EIA but not the HIV-Wb.

To determine whether archived plasma reactivity to HIV proteins could have been the result of passive acquired anti-HIV antibody contained in clotting factor concentrate, we assayed several concentrates made between 1981 and 1984 for the presence of anti-HIV-1 activity. One factor VIII concentrate from each of the 4 different U.S. manufacturers and one PCC made by the manufacturer that supplied over 90% of the PCCs used by our patient population were analyzed. Treatment with reconstituted clotting factor concentrate resulted in an approximate 1:100 dilution upon infusion into the blood stream (50 ml of reconstituted concentrate into 5 liters of whole blood). Accordingly, clotting factor concentrates were first diluted 1:100 prior to being run at the standard dilution for analysis (1:100 for HIV-Wb and 1:1.25 for the HIV-EIA). Under these parameters no anti-HIV reactivity was detectable in any of the clotting factor concentrates that were tested (data not shown). Additional studies in which clotting factor concentrates were spiked with limiting dilutions of HIV-1 specific antibodies were reactive indicating that the failure to detect anti-HIV antibodies was not due to interference by other clotting factor concentrate components (data not shown). Using more concentrated reconstituted clotting factor concentrates (a total dilution of 1:50) we could detect some HIV-Wb reactivity in all four of the factor VIII concentrates tested. This reactivity was with the p17, p24 and gp160 viral antigens, which confirms that CFCs were contaminated with blood products from HIV-seropositive donors (data not shown). Reactivity to the p17 and p160 viral proteins was rarely observed in the indeterminate samples from our archived plasma sets. This pattern of HIV-Wb reactivity would have been better represented in our archived plasma sets if the observed reactivity resulted from passively acquired anti-HIV antibodies present in contaminated clotting factor concentrates and introduced to the patient at the time of CFC transfusion. HIV-1-negative individuals (2.4%) had a slightly higher incidence of homozygosity for the deletion mutation of CCR5 compared with HIV-1-positive subjects (0%). These values reflect essentially those observed in the normal population as well as those in large cohort hemophiliac populations published earlier [11-14,16]. In this study there was no apparent protective advantage of CCR-5Δ32 heterozygosity in terms of HIV infection as has been reported previously [15-18].

## Conclusion

Our results suggest that some severe hemophiliacs who were repeatedly exposed to CFCs contaminated with infectious and/or non-infectious HIV-1, immunologically processed some of the viral antigens but were not infected. It is expected that some of the HIV proteins in CFCs that the hemophiliacs in our study were exposed to would have been associated with non-infectious particles. However, we feel it likely that some of these individuals were transiently infected with HIV and then cleared the infection. The anti-HIV humoral reactivity that we detected would appear to be insufficient to abort a viral infection but the lack of any archived PBMC make it impossible to assess the degree to which any of our hemophiliacs may have mounted an anti-HIV cellular immune response at the time of exposure. It is also possible that some of the hemophiliacs in our study may have been exposed to an immunizing, but not infectious doses of HIV.

## Methods

### Patients

Archived plasma samples from 15 HIV seronegative individuals with either hemophilia A or B were selected for this study. All had been regularly followed by the Louisiana Comprehensive Hemophilia Care Center at Tulane University School of Medicine. Patient characteristics and clotting factor concentrate exposure history are given in table 1. Patients were chosen for study based upon use of clotting factor concentrates in excess of 5000 units per year from 1979 to 1985 and on availability of archived plasma samples for retrospective testing. At initial testing with first generation HIV enzyme linked immunosorbent assays (ELISA) in 1985 all of the individuals included in the present study were categorized as HIV seronegative. All studies described below were performed on samples of citrated plasma that had been stored at -70°C.

### Detection of antibodies to HIV

#### HIV-1AB HIV-1IHIV-2 (rDNA) EIA

Archived plasma samples were reassessed for the presence of plasma antibodies reactive with HIV-1 and/or HIV-2 antigens using the HIV-1AB HIV-1IHIV-2 (rDNA) EIA kit (HIV-BIA, Abbott Laboratories) and HIV-1 and HIV-2 Wb assays (Cambridge Biotech, Rockville, Maryland) according to the manufactures protocol. In brief, the HIV-EIA is a current generation ELISA (1992) that utilizes a polystyrene bead coated with recombinant HIV-1 Env and Gag peptides and HIV-2 Env peptides. Test or control plasma was incubated at a dilution of 1:1.25 with viral antigen coated beads. Following washing, HIV reactive antibodies were detected by incubation of the bead-antibody complex with horseradish-peroxidase labeled HIV-1 and HIV-2 peptides that bind to all available open F'Ab sites. The enzyme-peptide-antibody complex was detected using a colorometric developing substrate solution comprised of 0-phenylene diamine~ 2HCl and analyzed spectrophotometrically at a wavelength of 492 nm. The frequency of reactivity on the HIV-EIA when tested on random blood donors is 0.16%. (HIV-1AB HIV-1/HIV-2 (rDNA) EIA, manual 83-8291IR4, 1992. Abbott Laboratories).

#### HIV-1 and HIV-2 Wb

HIV-1 and HIV-2 Wb analysis was performed using nitrocellulose strips containing electrophoretically separated and transferred proteins from inactivated HIV-1 or HIV-2 lysates. HIV-1 and HIV-2 Wb strips were subsequently incubated with test or control plasma at a dilution of 1:100. HIV reactive antibodies were visualized using biotinylated goat-anti human IgG and IgM, avidin-conjugated horseradish peroxidase and the colorimetric substrates 4-chloro-l-naphthol. Results on the HIV-1 Wb were classified as negative if no bands are present, and positive if any two or more of the following bands were present; p24, gp4l, gp120 and gp160 and had a reactivity score equal to or greater than the weak positive control. Indeterminate classification was given when banding was present, but did not meet the criteria for a positive interpretation [55]. The frequency of seropositivity on the HIV-l Wb when tested on random blood donors is 0.15% (HIV-1 Cambridge Biotech HIV-1 Western blot kit package insert).

### Passive HIV reactive antibodies in clotting factor concentrates

We assayed four factor VIII and one prothrombin complex concentrate (PCC) each manufactured between 1981 and 1984 for the presence of HIV reactive antibodies. All concentrates had been stored at 4°C in their original sealed vials in a lyophilized state. Each was reconstituted for the present study according to the manufacturer's instructions using sterile water. Despite their age, all were readily reconstituted and had a normal appearance. Reconstituted concentrates were immediately aliquoted into l ml cryovials and placed at -70°C until their use [56]. To assess HIV reactivity, reconstituted factor concentrates were diluted 1:1.33, 1:10, *1:50*, and 1:250 for HIV-1/HIV-2 RIA analysis and 1:50, 1:100, 1:200, 1:400, 1:1,000, 1:5,000, and 1:10,000 for HIV-1 Wb analysis. To determine the degree to which other components in clotting factor concentrates may interfere with the EIA or the Wb, a factor concentrate positive control was made by spiking aliquots of reconstituted clotting factor concentrates manufactured post 1990 with HIV-1 positive control sera and assayed for reduced reactivity.

### Determination of CCR-5 Genotype by PCR-RFLP

Whole blood was lysed in RBC lysis solution (DNA isolation kit, Gentra Systems) for 1 minute at room temperature. Lysates were then centrifuged for 20 seconds at 13,000–16,000*g and the supernant was removed. Cell pellets were vortexed in residual liquid and 300 μl of cell lysis solution was added. CCR-5 genotyping was determined using PCR-RFLP as has been previously described.

### Confirmation of HIV infection status

Due to the current HIV seronegative status of the hemophiliac population that we retrospectively analyzed in this study, it was considered unlikely that any were currently sub-clinically infected with HIV. However, to confirm this, recent peripheral blood mononuclear cell samples were assessed for the presence of HIV proviral DNA using HIV-1 PCR analysis.

## Competing interests

The author(s) declare that they have no competing interests

## Authors' contributions

SAT designed and performed the EIA and Wb experiments, analyzed the data and wrote the manuscript. Chemokine receptor PCR-RFLP was performed and analyzed by CAM and HEM. SSA provided intellectual assistance with Wb interpretation. RFG and CAL oversaw the design, development, implementation and analysis of the data for the project and edited the manuscript.
